# Prevalence of early and late menopause and its determinants in Rafsanjan cohort study

**DOI:** 10.1038/s41598-023-28526-y

**Published:** 2023-02-01

**Authors:** Hajar Vatankhah, Parvin Khalili, Mahboubeh Vatanparast, Fatemeh Ayoobi, Ali Esmaeili-Nadimi, Zahra Jamali

**Affiliations:** 1 Clinical Research Development Unit (CRDU), Niknafs Hospital, Rafsanjan University of Medical Sciences, Rafsanjan, Iran; 2 Department of Obstetrics and Gynecology, School of Medicine, Rafsanjan University of Medical Sciences, Rafsanjan, Iran; 3 Social Determinants of Health Research Center, Rafsanjan University of Medical Sciences, Rafsanjan, Iran; 4 Department of Epidemiology, School of Public Health, Rafsanjan University of Medical Sciences, Rafsanjan, Iran; 5 Molecular Medicuine Research Center, Research Institute of Basic Medical Sciences, Rafsanjan University of Medical Sciences, Rafsanjan, Iran; 6 Non-Communicable Diseases Research Center, Rafsanjan University of Medical Sciences, Rafsanjan, Iran

**Keywords:** Medical research, Risk factors

## Abstract

Our investigation aimed to evaluate the prevalence of early and late menopause and its determinants in adult women of Rafsanjan cohort study. We used data obtained from the Rafsanjan Cohort Study, as a part of the prospective epidemiological research studies in Iran. In this cross-sectional research, 2002 postmenopausal women were included in the present study. Menopause age were divided into three groups (**≤ **41 years, 42–54 years, and ≥ 55 years) based on the 10th and 90th percentile. The association between age at menopause with demographic and reproductive characteristics and some clinical risk factors of women was evaluated by logistic regressions. The mean age at menopause among the study participants was 48.63 ± 5.37 years. In this study, 11.49% and 11.39% of the women experienced early and late menopause respectively. After adjusting for all potential confounders, the results showed that taller and smoker women had higher odds of early menopause (OR 1.03, 95% CI 1.00–1.06) and OR 1.85, 95% CI 1.01–3.41) respectively) and women with history of using hormonal contraceptive more than median had lower odds of early menopause (OR 0.61, 95% CI 0.41–0.91). Also older women (OR 8.65, 95% CI 5.31–14.08) and women with a history of diabetes (OR 2.42, 95% CI 1.63–3.60), hypertension (OR 2.06, 95% CI 1.42–2.97), thyroid disease (OR 1.85, 95% CI 1.07–3.20) and depression (OR 2.00, 95% CI 1.35–2.97) had higher odds of late menopause. The results showed that the year of birth, height, smoking, history of diabetes, hypertension, thyroid disease and depression and using hormonal contraceptive were significantly associated with the menopausal age. Since age at menopause can affect subsequent health in women, understanding the determinants of menopausal age is important and should be pursued.

## Introduction

Menopause is an important event in the woman’s life that occurs due to the suppression of ovarian function and leads to the end of the reproductive period^1^. The median age of menopause in women is 51 years, and approximately 4% of women experience menopause before the age of 40 years^2^. It has been reported that women in developing countries experience menopause earlier than women in developed countries^3^. A review study reported that the median age of menopause in Iran was 48.57 years with the maximum and minimum ages of 52.00 and 46.34 years respectively^4^. Previous studies have shown that menopause can be influenced by genetic factors, obesity, alcohol consumption, social status, ethnicity, education, diet, exposure to pesticides, reproductive factors^5^, and area of residence^3^ but the results are inconsistent. Recently, special attention has been paid to the age at menopause because it can affect subsequent health in women^6^. There is evidence that early menopause increases the risk of cardiovascular disease, atherosclerosis, stroke, osteoporosis^6^ and alzheimer's^7^. On the other hand, late menopause was associated with an increased risk of breast cancer, ovarian and endometrial cancers^8^. Therefore, identifying the factors associated with early and late menopause, especially those that are modifiable, may be very important to prevent these chronic diseases. According to some authors, menopausal age is considered as a health indicator^9^. Thus, a better understanding of this issue can improve the life quality of middle-aged women. This study aimed to determine the mean age at menopause and identify the factors related to early and late menopause in adult women of Rafsanjan cohort study, with the hope that the results would promote the knowledge regarding the risk factors of these phenomena.

## Methods

This cross-sectional study was carried out based on the data of Rafsanjan Cohort Study (RCS), as part of the Prospective Epidemiological Research Studies in Iran (PERSIAN)^10^ which was conducted in Rafsanjan, one of the cities located in the southeast of Iran. RCS is a population-based prospective cohort launched in August 2015. The enrollment phase ended in December 2017, and a 15-years follow-up is planned. At the baseline study, trained questioners collected data on socio-demographic and anthropometric measurements, medical history, lifestyle, nutrition, reproductive characteristics of participants using a laptop-based questionnaire. All participants provided written informed consent. Study protocol was approved by the ethics committee of Rafsanjan University of Medical Sciences (Ethical codes: ID: IR.RUMS.REC.1400.142). Detailed information about the study design, methods and recruitment procedures have been described previously^11^. In this study, 9991 participants, including 5336 women aged 35–70 years were recruited. Among them, 2359 women were postmenopausal and reported valid data of menopause age. Women who had a history of cancer and those who had history of hysterectomy or ovariectomy (unilateral or bilateral) before menopausal age were excluded from the study. Finally, 2002 postmenopausal women were included in the present study.

Socio-economic status was measured according to the Wealth score index (WSI). WSI was estimated by multiple correspondence analysis (MCA) of the variables such as access to a dish washer, access to a computer, access to a car, the price of the car, owning a mobile, number of international trips in lifetime. Marriage status was dichotomized as married and single status including never married, divorced, widowed or other. Smoking status (having smoked at least 100 cigarettes during lifetime: yes, no) and opium usage (having used opium at least once per week for 6 months: yes, no) were self-reported. The level of physical activity was calculated based on metabolic equivalent task hours (MET-hours/day). Women that reported diagnosis age of diabetes, hypertension, depression, myocardial infarction (MI), cardiac diseases, fatty liver and thyroid disease by a physician before menopausal age were considered as positive history of these diseases. Year of women birth categorized into three groups based on the 25th and 50th percentile.

Menopause age (≤ 41 years, 42 ± 54 years, and ≥ 55 years) and menarche age (**≤ **12 years, 13–15 years, and ≥ 16 years) were divided into three groups based on the 10th and 90th percentile. History of using hormonal contraceptive (oral contraceptive pills (OCP), implants and long-term progesterone injection) was divided into three groups as never used, ≤ median and > median in users. Other reproductive characteristics, including first marriage age, age at first pregnancy, age at first delivery, number of live children and total breastfeeding duration were divided into two groups based on median. Age at first pregnancy, number of live children and total breastfeeding duration were asked in women with at least one pregnancy. Age at first delivery was asked in women with at least one live child. The frequency difference between total number and some of the variables was related to missing data. Accuracy and precision of all methods were performed in accordance with the relevant guidelines and regulations.

### Statistical analysis

The series of characteristics of individuals were compared across the groups of menopausal age (normal, early, elevated) using chi-square test for categorical variables and the Kruskal–Wallis test for non-normally distributed quantitative variables. Quantitative variables were described as the median (IQR) and categorical variables as the frequency and percentage.

We used multinomial logistics regression models to determine the odds ratios (ORs) and the corresponding 95% confidence intervals (CI) for the association between menopause age and selected characteristics compared to the menopausal age at 42–54 years as the reference. Potential confounding variables were sequentially entered into model according to their hypothesized strengths of association with menopausal age. Variables with a *p*-value < 0.25 were selected as confounders. The adjusted model included for year of birth (categorical variable), height (continuous variable), waist (continuous variable), wealth status index (continuous variable), education years (continuous variable), physical activity level (continuous variable), has job (yes, no), cigarette smoking (yes/no), diabetes (yes/no), hypertension (yes/no), thyroid disease (yes/no), cardiac diseases (yes/no), depression (yes/no), menarche age (continuous variable), first marriage age (continuous variable), number of live children (continuous variable), breastfeeding duration (continuous variable) and using hormonal contraceptive (categorical). All analyses were performed using State V.12. All *p*-values are two-sided, and *p*-values < 0.05 and 95% confidence intervals were considered as statistically significant.

### Ethics approval and consent to participate

The ethics committee of Rafsanjan University of Medical Sciences approved this study (Ethical codes: ID: IR.RUMS.REC.1400.142). Written informed consent was obtained from the participants. The data of Participants kept confidential and was only accessible to the study investigators.

## Results

The mean age at menopause among the study participants was 48.63 ± 5.37 years. In this study, 11.49% (n: 230) and 11.39% (n: 228) of the women experienced early and late menopause respectively. Table 1 shows some selected characteristics including socio-demographic, anthropometric measures, personal habits and clinical risk factors stratified by age at menopause. We observe significant differences between the three menopausal age groups with year of birth, WSI, education, job, history of diabetes, hypertension, cardiac diseases, MI and depression (Table 1, *p* < 0.05). The frequency of women with early menopause increased and frequency of women with late menopause decreased across year of birth from ≤ 1953 until ≥ 1958.

**Table 1:** Demographic characteristics of women according to age at menopause in Rafsanjan Cohort Study (RCS).

Variable	Total (n = 2002)	Menopause age			
		≤ 41 (n = 230)	42–54 (n = 1544)	≥ 55 (n = 228)	p-value
Year of birth- n. (%)					< 0.001
≤ 1953	495 (24.73)	45 (19.57)	346 (22.41)	104 (45.61)	
1954–1957	470 (23.48)	50 (21.74)	333 (21.57)	87 (38.16)	
≥ 1958	1037 (51.80)	135 (58.70)	865 (56.02)	37 (16.23)	
WSI					0.027
Median (IQR)	− 0.51 (− 1.04–0.48)	− 0.17 (− 0.98–0.48)	− 0.44 (− 0.98–0.48)	− 0.51 (− 1.15–0.08)	
BMI.kg/m^2^				0.429	
Median (IQR)	29.49 (26.32–32.55)	28.97 (26.3–32.38)	29.50 (26.3–32.46)	29.90 (26.56–33.02)	
Weight.kg				0.98	
Median (IQR)	70.5 (63–79)	70 (63–79)	70.5 (63–78.5)	71 (62.5–80)	
Height.cm					0.12
Median (IQR)	154.6 (151.1–158.3)	155.2 (151.7–158.9)	154.6 (151–158.35)	154 (150.75–158)	
Waist.cm					0.234
Median (IQR)	100.6 (93.9–107.1)	100.02 (94–107)	100.06 (93.8–107)	101.06 (95–108.25)	
Education. Year					< 0.001
Median (IQR)	5 (0–8)	5 (1–9)	5 (1–8)	2 (0–5)	
Physical activity					0.214
Median (IQR)	37.35 (35.25–39.4)	37.53 (35.48–39.48)	37.33 (35.25–39.4)	37.13 (34.73–39.15)	
Has Job- no. (%)					0.005
Yes	186 (9.30)	26 (11.30)	152 (9.85)	8 (3.52)	
No	1814 (90.70)	204 (88.70)	1391 (90.15)	219 (96.48)	
Residence type- no. (%)					0.076
Urban	1699 (84.87)	189 (82.17)	1306 (84.59)	204 (89.47)	
Rural	303 (15.13)	41 (17.83)	238 (15.41)	24 (10.53)	
Smoking-no. (%)				0.085	
Yes	86 (4.30)	15 (6.52)	58 (3.76)	13 (5.70)	
No	1914 (95.70)	215 (93.48)	1484 (96.24)	215 (94.30)	
Opium consumption- no. (%)					0.796
Yes	129 (6.44)	15 (6.52)	97 (6.28)	17 (7.46)	
No	1873 (93.56)	215 (93.48)	1447 (93.72)	211 (92.54)	
History of diabetes- no. (%)					< 0.001
Yes	256 (12.79)	9 (3.91)	190 (12.31)	57 (25)	
No	1746 (87.21)	221 (96.09)	1354 (87.69)	171 (9.79)	
History of hypertension—no. (%)					< 0.001
Yes	355 (17.73)	9 (3.91)	276 (17.88)	70 (30.70)	
No	1647 (82.27)`	221 (96.09)	1268 (82.12)	158 (69.30)	
History of MI—no. (%)					0.023
Yes	16 (0.80)	0 (0)	11 (0.71)	5 (2.19)	
No	1986 (99.20)	230 (100)	1533 (99.29)	223 (97.81)	
History of cardiac ischimic—no. (%)					0.021
Yes	77 (3.85)	2 (0.87)	62 (4.02)	13 (5.70)	
No	1925 (96.15)	228 (99.13)	1482 (95.98)	215 (94.30)	
History of depression- no. (%)					0.001
Yes	345 (17.23)	22 (9.57)	271 (17.55)	52 (22.81)	
No	1657 (82.77)	208 (90.43)	1273 (82.45)	176 (77.19)0	
History of fatty liver- no. (%)					0.434
Yes	46 (2.30)	3 (1.30)	39 (2.53)	4 (1.75)	
No	1956 (97.70)	227 (98.70)	1505 (97.47)	224 (98.25)	
History of thyroid disease- no. (%)					0.258
Yes	146 (7.29)	12 (5.22)	114 (7.32)	21 (9.21)	
No	1856 (92.71)	218 (94.78)	1431 (92.68)	207 (90.79)	

*WSI* Wealth score index, *BMI* body mass index, *MI* myocardial infarction.

The reproductive characteristics of participants stratified by age at menopause are shown in Tables 2. The median of pregnancy number, number of live children, history of infertility, total duration of breastfeeding and history of using hormonal contraceptive had significant relationships with menopausal age groups (Table 2, *p* < 0.05). The frequency of pregnancy number > median, live children number > median, positive history of infertility, breastfeeding duration > median and history of using hormonal contraceptive > median was significantly higher among women with late menopause compared to other menopausal age groups.

**Table 2:** Reproductive characteristics of women according to age at menopause in Rafsanjan Cohort Study (RCS).

Variable	Total (n = 2002)	Menopause age			
		Early: ≤ 41 (n = 230)	Normal: 42–54 (n = 1544)	Late: ≥ 55 (n = 228)	*p*-value
Menarche age no. (%)					0.226
≤ 12	476 (23.78)	57 (24.78)	371 (24.03)	48 (21.05)	
13–15	1314 (65.63)	144 (62.61)	1022 (66.19)	148 (64.91)	
≥ 16	212 (10.59)	29 (12.61)	151 (9.78)	32 (14.04)	
Median (IQR)	14 (13–15)	14 (13–15)	14 (13–15)	14 (13–15)	0.18
Marital status no. (%)				0.275	
Single	391 (19.53)	47 (20.43)	291 (18.85)	53 (23.25)	
Married	1611 (80.47)	183 (79.57)	1253 (81.15)	175 (76.15)	
First marriage age-no. (%)				0.277	
≤ Median	1131 (56.72)	129 (56.33)	862 (56.05)	140 (61.67)	
> Median	863 (43.28)	100 (43.67)	676 (43.95)	87 (38.33)	
Median (IQR)	19 (17–22)	19 (17–22)	19 (17–22)	18 (16–21)	0.06
Age at first pregnancy-no. (%)				0.550	
≤ Median	1148 (58.45)	136 (60.99)	875 (57.79)	137 (60.35)	
> Median	816 (41.55)	87 (39.01)	639 (42.21)	90 (39.65)	
Median (IQR)	20 (18–22)	20 (17–22)	20 (18–22)	20 (17–22)	0.246
Number of Pregnancy-no. (%)				< 0.001	
≤ Median	1246 (62.39)	157 (68.26)	997 (64.78)	92 (40.35)	
> Median	751 (37.61)	73 (31.74)	542 (35.22)	136 (59.65)	
Median (IQR)	6 (4–8)	5 (3–7)	6 (4–7)	7 (5–9)	< 0.001
Age at first delivery-no. (%)				0.355	
≤ Median	1152 (59.05)	139 (62.61)	874 (58.19)	139 (61.23)	
> Median	799 (40.95)	83 (37.39)	628 (41.81)	88 (38.77)	
Median (IQR)	21 (18–23)	21 (18–23)	21 (19–23)	20 (18–23)	0.265
History of abortion-no. (%)				0.643	
Yes	769 (39.15)	81 (36.32)	597 (39.43)	91 (40.09)	
No	1195 (60.85)	142 (63.68)	917 (60.57)	136 (59.91)	
Number of live children-no. (%)				< 0.001	
≤ Median	1150 (58.55)	144 (64.57)	918 (60.63)	88 (38.77)	
> Median	814 (41.45)	79 (35.43)	596 (39.37)	139 (61.23)	
Median (IQR)	5 (4–7)	5 (3–6)	5 (4–6)	6 (5–8)	< 0.001
History of stillbirth-no. (%)				0.660	
Yes	225 (11.46)	26 (11.66)	169 (11.16)	30 (13.22)	
No	1739 (88.54)	197 (88.34)	1345 (88.84)	197 (86.78)	
History of infertility, no. (%)				0.028	
Yes	324 (16.22)	43 (18.70)	232 (15.07)	49 (21.49)	
No	1673 (83.78)	187 (81.30)	1307 (84.93)	179 (78.51)	
History of using infertility drugs, no. (%)				0.574	
Yes	95 (4.76)	13 (5.65)	69 (4.48)	13 (5.70)	
No	19.02 (95.24)	217 (94.35)	1470 (95.52)	215 (94.30)	
Tubectomy no. (%)				0.399	
Yes	666 (33.27)	73 (31.74)	525 (34)	68 (29.82)	
No	1336 (66.73)	157 (68.26)	1019 (66)	160 (70.18)	
Total duration of breastfeeding -no. (%)				< 0.001	
≤ Median	992 (50.51)	124 (55.61)	791 (52.25)	77 (33.92)	
> Median	972 (49.49)	99 (44.39)	723 (47.75)	150 (66.08)	
Median (IQR)	78 (48–120)	72 (40–108)	76 (46–114)	102 (60–144)	< 0.001
History of using hormonal contraceptive, no. (%)				0.186	
No	1084 (54.15)	133 (57.83)	831 (53.82)	120 (52.63)	
≤ Median in users	460 (22.98)	59 (25.65)	349 (22.60)	52 (22.81)	
> Median in users	458 (22.88)	38 (16.52)	364 (23.58)	56 (24.56)	
Median (IQR) in users	34 (12–72)	12 (2–36)	24 (3–60)	24 (6–72)	0.026

Table 3 presents the association of early and late menopause with the selected variables using multinomial logistics regression model. As seen in this table, height and smoking were associated with higher odds of early menopause (OR 1.03, 95% CI 1.00–1.06 and OR 1.85, 95% CI 1.01–3.41 respectively). Women with history of diabetes, hypertension and thyroid disease had lower odds of early menopause by 64%, 76% and 51% respectively (OR 0.36, 95% CI 0.17–0.76; OR 0.24, 95% CI 0.12–0.48; OR 0.49 95% CI 0.30–0.80). Also, using hormonal contraceptive > median was related to decreased odds of early menopause by 39% (OR 0.61, 95% CI 0.41–0.91). We observed significant association between the increased odds of late menopause with year of birth, history of diabetes, hypertension and thyroid disease and depression. The odds of late menopause was highest among women with year of birth ≤ 1953 (OR 8.65, 95% CI 5.31–14.08). The odds of late menopause among women born during 1954–1957, was 7.14 (95% CI 4.56–11.18).

**Table 3:** The association of early and late menopause with the selected variables using multinomial logistics regression model.

Variables	Adjusted model (OR (95% CI))	
	Early menopause	Late menopause
Year of birth		
≤ 1953	0.77 (0.50–1.17)	8.65 (5.31–14.08)
1954–1957	0.91 (0.62–1.32)	7.14 (4.56–11.18)
≥ 1958	1	1
Height	1.03 (1.00–1.06)	1.01 (0.98–1.04)
Waist	1.00 (0.99–1.01)	1.01 (0.99–1.02)
WSI	1.02 (0.82–1.20)	1.06 (0.89–1.26)
Education	0.99 (0.95–1.03)	0.98 (0.954–1.03)
Physical activity	1.01 (0.96–1.05)	1.03 (0.98–1.07)
Job	0.98 (0.61–1.58)	0.53 (0.25–1.16)
Smoking	1.85 (1.01–3.41)	0.74 (0.38–1.45)
Diabetes	0.36 (0.17–0.76)	2.42 (1.63–3.60)
Hypertension	0.24 (0.12–0.48)	2.06 (1.42–2.97)
Thyroid disease	0.80 (0.42–1.50)	1.85 (1.07–3.20)
Cardiac diseases	0.34 (0.08–1.43)	1.63 (0.80–3.31)
Depression	0.49 (0.30–0.80)	2.00 (1.35–2.97)
Menarche age	1.02 (0.93–1.11)	1.04 (0.95–1.14)
First marriage age	0.97 (0.93–1.01)	1.02 (0.97–1.06)
Number of live children	0.99 (0.89–1.10)	1.07 (0.97–1.19)
Breastfeeding duration	1.00 (0.99–1.01)	1.00 (1.00–1.01)
Using hormonal contraceptive		
0	1	1
≤ Median	1.01 (0.71–1.43)	1.08 (0.74–1.58)
> Median	0.61 (0.41–0.91)	1.13 (0.78–1.63)

The adjusted model is adjusted for confounding variables: year of birth (categorical variable), height (continuous variable), waist (continuous variable), wealth status index (continuous variable), education years (continuous variable), physical activity level (continuous variable), has job (yes, no), cigarette smoking(yes/no), diabetes (yes/no), hypertension (yes/no), thyroid disease (yes/no), cardiac diseases (yes/no), depression (yes/no), menarche age (continuous variable), first marriage age (continuous variable), number of live children (continuous variable), breastfeeding duration (continuous variable) and using hormonal contraceptive (categorical).

## Discussion

The results of this cross sectional study of 2002 postmenopausal women in Rafsanjan showed that the mean age at menopause is 48.63 years, which is in accordance with many studies that have reported a mean age of 48 years for menopause age in Iranian communities^12^. Iranian women reached the menopause earlier in comparison to the other parts of geographic regions such as Europe (50.1–52.8) and North America (50.5–51.4). But the menopause age of our population was more than Middle East (46.9–47.8) and Latin America (45.9–41.6)^13^. An interesting finding in the study of some selected variables related to menopause age was that late menopause decreased across year of birth from ≤ 1953 until ≥ 1958. It was found that year of birth can be an important determinant of age at menopause. The older women (birth year ≤ 1953) were accompanied with more chance to experience menopause at the older age (8.65 times). This data is in contrast with the study of Gottschalk et al. in Norway population which has reported an increase in the age at menopause from 50.31 among women born during 1936–1939 to 52.73 among women born during 1960–1964^14^.

Also in study of Northeast et al. in China, the early menopause was more prevalent in subjects born during 1943–1947 (OR 1.708, 1.205–2.420) and 1933–1937 (OR = 2.445, 1.525–3.921) compared with the reference group (born from 1948 to 1952)^15^. The lower age at menopause not exactly reflect increasing in the incidence of earlier menopause, but it is an alarm for women health. The early menopause following estrogen deficiency besides premature loss of fertility was associated with subsequent health problems such as osteoporosis, cardiovascular disease, sexual dysfunction, neurologic outcomes, cancers and mortality^7,16^. Early menopause may be a result of an accelerated aging process due to genetic or non-genetic causes^16^. The variation in menopausal age may be due to the differences in race, lifestyle behavior, area of residence, diet, reproductive characteristics^8^ and social and economic factors^13^. Some differences in the levels of endogenous hormones in Asian population compared to Western women reflected the role of ethnicity^17^.

The results showed that taller women had more odds for the early menopause (OR: 1.03(1.00–1.06)). Inconsistent with our results, there was a significant trend toward late age at menopause in taller women among Yemen women^18^. However in some previous studies the authors reported that there was no relationship between height and the age at menopause^19^. Our results also showed an increased odds of early menopause among smoker women (1.8 times). It’s in accordance with the other studies that showed smoking was correlated with the early onset of menopause^8^. The proposed mechanism is that nicotine accelerates the loss of ovarian follicles and affects converting androgen into estrogen by blocking the aromatase enzyme. Also it was demonstrated that the peak of the luteinizing hormone delayed in the middle of the cycle by inhalation of tobacco smoke^20^.

Interestingly in women with a history of diabetes, the odds of early menopause decreased by 64% (play a protective role) and the odds of late menopause increased about 2.5 times. In accordance with our results a positive relationship has been showed between the late menopause and diabetes in a previous study^15^. Also, Brand et al.^21^ reported that diabetes developed at a younger age (< 20 years) was associated with a lower age at menopause, while diabetes started at an older age (> 50 years) was associated with late menopause. It has been shown that premature vascular aging in Type 1 diabetes leads to the early depletion of ovarian follicle pool and causes early menopause^22^. However our finding is in contrast with the study of Sekhar et al. which reported type 2 diabetes increased the risk of lower age at menopause^23^.

The present study also showed that hypertension and thyroid disease decreased the odds of early menopause (by 76%, and 51% respectively). Bustami et al. reported that hypertension was associated with normal or late menopause^24^. However, in contrast with our result some studies showed that women with hypertension reach to menopause earlier^19,25^. In addition, thyroid has also been postulated to lower menopause age in a Iranian population^25^. Adjustment factors or other methodological differences may explain this controversy.

Our results showed that women with a history of depression had more odds of late menopause. This finding is in agreement with the study of Li et al. that reported women with depression were associated with later menopause compared to women with no such history^26^. The authors concluded that since depression is common among women with PCOS (polycystic ovary syndrome), possibly the same biological mechanisms causes delayed menopause in women with PCOS and depression^26^. But inconsistent results have been reported in other studies that concluded medically treated depression was associated with early menopause^27^.

The results of this study showed that the women who had used hormonal contraceptive more than median, had lower odds of early menopause (by 39%). While, in study of Aydin et al. earlier menopause was associated with longer years on oral contraceptive, in multivariate analysis^19^. In agreement with our study, a systematic review and meta-analysis study showed that use of oral contraceptives before menopause was associated with later menopause^28^. One physiologic reason for this association may be that oral contraceptives suppress the FSH level, and consequently delay age at menopause^29^.

The strengths of the present study included the large sample size and comprehensive evaluation of various factors on the menopausal age among women. Therefore, a major strength of our investigation has been adjustment of different potential confounders such as demographic information, history of diseases and reproductive characteristics. Nonetheless, there were a number of limitations in our research. First, the data of outcome and exposure variables were collected mainly based on self-reported and therefore some women may have been misclassified and may be subject to reminder bias. However, previous studies on reproducibility of self-reported age at natural menopause have shown that menopausal age by recall is reasonably well^30^. Second, due to cross-sectional design of study, we could not provide causal association between the various factors and age at menopause. Further longitudinal studies are needed to clarify these associations.

## Conclusion

This study showed that taller and smoker women had higher odds of early menopause. Also older women, women with history of using hormonal contraceptive and women with a history of diabetes, hypertension, thyroid disease and depression had higher odds of late menopause. Since age of menopause can affect subsequent health in women, understanding the determinants of menopausal age is important and should be pursued.

## Data Availability

The datasets used during the current study are available on the Persian Adult Cohort Study Center, Rafsanjan University of Medical Sciences, Iran. The data is not available publicly. However, upon a reasonable request, the data can be obtained from the corresponding author.
